# Bioactive Compounds from Marine Sponges: Fundamentals and Applications

**DOI:** 10.3390/md19050246

**Published:** 2021-04-27

**Authors:** Disha Varijakzhan, Jiun-Yan Loh, Wai-Sum Yap, Khatijah Yusoff, Rabiha Seboussi, Swee-Hua Erin Lim, Kok-Song Lai, Chou-Min Chong

**Affiliations:** 1 Aquatic Animal Health and Therapeutics Laboratory, Institute of Bioscience, Universiti Putra Malaysia, Serdang 43400, Malaysia; dishavarijakzhan@gmail.com; 2 Faculty of Applied Sciences, UCSI University, No. 1, Jalan Menara Gading, UCSI Heights, Cheras, Kuala Lumpur 56000, Malaysia; lohjy@ucsiuniversity.edu.my (J.-Y.L.); wsyap@ucsiuniversity.edu.my (W.-S.Y.); 3 Department of Microbiology, Faculty of Biotechnology and Biomolecular Sciences, Universiti Putra Malaysia, Serdang 43400, Malaysia; kyusoff@upm.edu.my; 4 Health Sciences Division, Al Ain Men’s College, Higher Colleges of Technology, Al Ain 17155, United Arab Emirates; rseboussi@hct.ac.ae; 5 Health Sciences Division, Abu Dhabi Women’s College, Higher Colleges of Technology, Abu Dhabi 41012, United Arab Emirates; lerin@hct.ac.ae; 6 Department of Aquaculture, Faculty of Agriculture, Universiti Putra Malaysia, Serdang 43400, Malaysia

**Keywords:** marine sponge, bioactive compounds, microbial symbionts, antimicrobial activity, aquaculture, quorum quenching

## Abstract

Marine sponges are sessile invertebrates that can be found in temperate, polar and tropical regions. They are known to be major contributors of bioactive compounds, which are discovered in and extracted from the marine environment. The compounds extracted from these sponges are known to exhibit various bioactivities, such as antimicrobial, antitumor and general cytotoxicity. For example, various compounds isolated from *Theonella swinhoei* have showcased various bioactivities, such as those that are antibacterial, antiviral and antifungal. In this review, we discuss bioactive compounds that have been identified from marine sponges that showcase the ability to act as antibacterial, antiviral, anti-malarial and antifungal agents against human pathogens and fish pathogens in the aquaculture industry. Moreover, the application of such compounds as antimicrobial agents in other veterinary commodities, such as poultry, cattle farming and domesticated cats, is discussed, along with a brief discussion regarding the mode of action of these compounds on the targeted sites in various pathogens. The bioactivity of the compounds discussed in this review is focused mainly on compounds that have been identified between 2000 and 2020 and includes the novel compounds discovered from 2018 to 2021.

## 1. Introduction

The open oceans and deep seas cover about 70% of the earth’s surface and are a natural habitat to approximately 80% of the world’s plant and animal species [1,2]. The ocean is known to accommodate various living organisms, ranging from prokaryotic bacteria and marine invertebrates to multicellular complex organisms such as sharks and whales [3]. Despite the extreme conditions of the marine environment, the accessibility of new compounds from marine organisms from the various depths of the sea is now possible due to the advancement in technologies such as scuba diving equipment, remotely operated vehicles, closed-circuit computerized mixed gas rebreathers and manned submersibles [4,5]. More than 5000 novel natural products have been extracted from marine organisms living in these extreme environments, having a wide thermal range from freezing temperatures to as high as 350 °C in deep hydrothermal vents, pressures ranging from 1 to 1000 atm, varying nutrient ranges and photic and non-photic zones [6,7]. 

**Figure 1:**
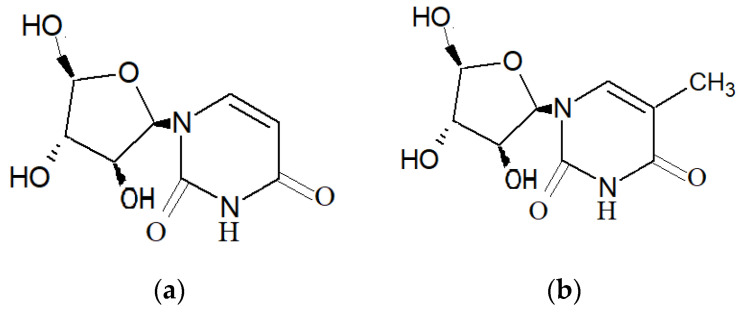
Chemical structures of (**a**) spongouridine and (**b**) spongothymidine [13].

**Figure 2:**
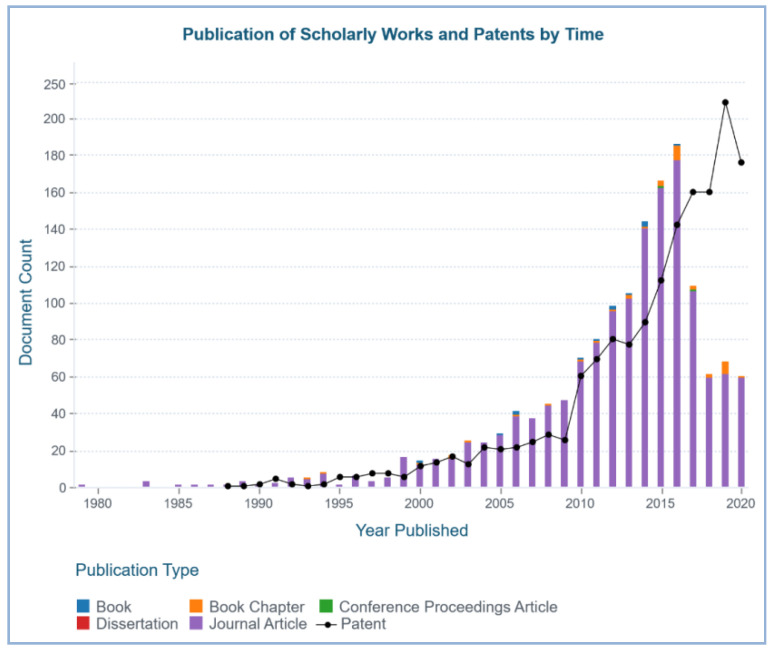
Publication of scholarly works (books, book chapters, journal articles, conference proceedings, dissertations) and patents that reported the discovery and/or applications of marine sponge–derived bioactive compounds retrieved from https://www.lens.org/ using keywords “marine sponge” and “bioactive compounds”.

Research involving natural compounds from marine organisms began in the 1950s with Bergmann and coworkers’ discovery of the compounds spongothymidine and spongouridine (Figure 1), extracted from the Caribbean sponge *Tectitethya crypta* (de Laubenfels, 1949) [4]. In the past few years, several marine taxa, such as sea slugs, sponges and soft corals, have been a major source of novel natural bioactive compounds [8,9,10]. From the initial stage of natural product marine research to the current trend of drug discovery, most of the natural compounds that have been discovered are from marine invertebrates, especially marine sponges, with hundreds of compounds discovered yearly from this source [3,11]. Figure 2 below showcases the data that were retrieved from https://www.lens.org/ using keywords “marine sponge” and “bioactive compounds”, illustrating the publication of scholarly works regarding the discovery of marine sponge–derived compounds and their application in the form of books, book chapters, journal articles, conference proceedings, dissertations and patents. A sponge is an invertebrate, sessile organism that can be widely found in temperate, tropical and polar habitats [4,12]. Marine sponges belong to the phylum Porifera, and there are more than 8000 species of sponges that have been discovered so far [11].

Marine sponges are made up of a jelly-like layer between two thin layers of cells and might contain the skeleton of spicules made of silica, calcium carbonate, and a protein known as spongin [14]. The identification of sponge species is based on the size and shape of the spicules [14]. Marine sponges are filter feeders; they cope with potentially hazardous particles by producing neutralizing bioactive compounds. Around 11 genera of sponges have been discovered to contribute to the discovery of bioactive compounds, including the three genera *Haliclona*, *Petrosia* and *Discodemia*, which are known to produce compounds with powerful anti-inflammatory and anti-cancer activities [7,15]. Bioactive compounds synthesized by sponges are chemically diverse and can be grouped as nucleosides, terpenes, sterols, cyclic peptides and alkaloids [11].This diverse range of bioactive compounds are not completely synthesized by sponges alone but are also synthesized by their microbial symbionts [3,14]. Table 1 below lists a few marine sponge species and their bioactivities, along with the chemical structure of the compounds.

In this review, we discuss the antimicrobial potential in human diseases of various bioactive compounds extracted from marine sponges and the application of these compounds in the aquaculture industry. The application of these compounds in other veterinary commodities is further discussed alongside the mode of action of these bioactive compounds on their targeted sites. In this review, we will be focusing on the compounds extracted from the extracts isolated during the period 2000 to 2020.

## 2. Ecological and Biological Factors Driving the High Abundance of Bioactive Compounds from Marine Sponges

Marine sponges have two different life cycles. At the initial stage, a larva is released from the female sponge into the water; it will eventually attach to a suitable substrate and will grow into a non-motile (sessile) adult [23]. Marine sponges are known as excellent filter feeders; the surrounding water is drawn into the sponge through small pores known as ostia, and the water is ejected through a larger opening known as osculum [24].

**Figure 3:**
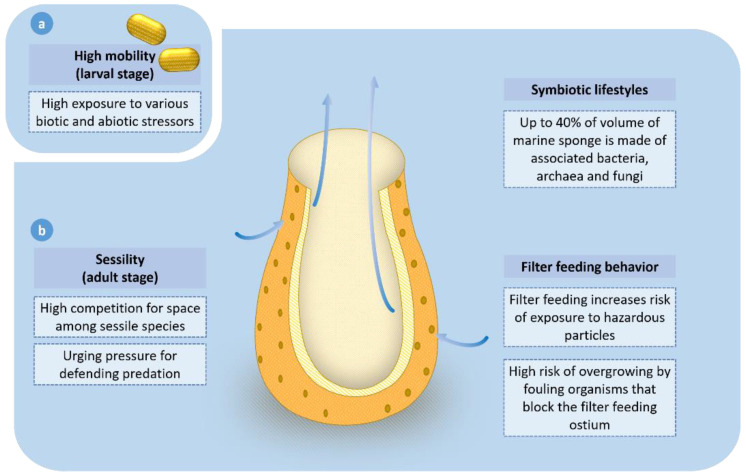
A summary of ecological and life-history-related factors that cause high production of bioactive compounds in marine sponges. (**a**) The larval stage is highly motile; hence, the larva is exposed to a high variety of pathogenic and abiotic stressors. (**b**) The non-motile adult experiences high competition for space among sessile organisms and predation by natural predators. Adults have symbionts attached to them. Filter feeding behavior of the adult increases the risk of exposure to hazardous materials. Overgrowing by fouling organisms like barnacles on the marine sponge has a lethal effect by blocking ostia. These factors have driven the high production of bioactive compounds in marine sponges.

There are specific cells present in the marine sponge that perform particular functions. Flagellated cells (choanocytes) allow water movement in one direction through the coordination of the flagella. Particulate matter present in the water is engulfed by the amoebocyte cells [25]. Figure 3 below depicts the ecological and life-history-related factors that drive the high production of bioactive compounds in marine sponges.

Microorganisms passed through the ostia remain in the mesohyl, which is the inner sponge of the tissue, thus forming a symbiotic interaction with the sponge. The symbionts consist mainly of bacteria but also archaea, eukaryotes or larger organisms [26]. The bacterial population found in the extracellular mesohyl matrix of the sponges harbors dense bacterial assemblages that could make up to 38% of the biomass of the sponge [23,27,28]. In some sponges, the bacterial density may even range up to 10^8^ to 10^10^ or 10^5^ to 10^6^ bacteria/g of the sponge’s wet weight [29]. Due to the difference in range between microbial population densities, marine sponges can be classified as “bacteriosponges” or “highly-microbial-abundance (HMA) and low-microbial-abundance (LMA)” sponges [23,29].

The ecological and evolutionary importance of the associations between sponge and microorganism can be determined by their ability to produce various bioactive compounds, which can be utilized in the biotechnology industry [30]. The advantage of secondary metabolites in the sponges can be identified based on the presence of antifouling products. For example, sponges can protect the water-pumping ability by preventing biofilm formation and the settlement of barnacles or bryozoans on their surface [31,32]. Sponges produce high-level cytotoxic chemicals by producing mucus-containing toxins, which create a clear zone around the sponge as a defense against other marine species. This ensures that sponges are able to conquer densely populated rocks or corals, enabling them to compete with organisms that grow rapidly. However, they are also able to selectively use these toxins without destroying themselves [30].

Secondary bioactive metabolites found in marine sponges are generally of microbial origin [33,34,35]. This can be proven through the structural similarity of some of the natural products that have been discovered from the sponges, such as complex polyketides and non-ribosomal peptides, which are exclusively known to microorganisms. However, some metabolites are produced by the sponges. For example, avarol is a compound found in the sponge *Dysidea avara*, stevensine is from *Axinella corrugate* (George and Wilson, 1919) and an array of cytotoxic brominated isoxazoline alkaloids are the natural products of the sponges themselves [36,37,38]. In addition, some enzymes with potential therapeutic applications have been isolated from sponges. For instance, ATP N-glycosidase is an enzyme discovered in *Axinella polypoides* (Schmidt, 1862) that converts adenosine-5′-tri-phosphate (ATP) into adenine and ribose-5-triphosphate [39]. The hydrolysis of ATP takes place by hydrolyzing the N-glycosidic bond that exists in the ATP molecule, thus leaving the energy-rich triphosphate moiety. The cleavage results in ribose-5-phosphate, which is utilized in the synthesis of nucleic acids deoxyribonucleic acid (DNA) or ribonucleic acid (RNA) [40].

## 3. Chemistry of Compounds from Marine Sponges

The extracts obtained from marine sponges consist of a collection of secondary metabolites that are synthesized by the sponges and also by the symbionts attached to the sponges [17,41]. These secondary metabolites synthesized by the sponges and the symbionts function as a defense mechanism; the synthesis of these metabolites depends on various stress factors, such as predators, overgrowth of fouling organisms and competition for space with other organisms [3,42]. These secondary metabolites are chemically diverse, and there are various factors that influence the compounds that can be extracted from the marine sponges [3,43]. For example, sponges from the genus *Haliclona* synthesize a wide range of metabolites ranging from class terpenes, such as sterols, sesquiterpenoid quinols, glycosphingolipids, and alkaloid bioactive secondary metabolites [44]. Table 2 below shows examples of compounds from marine sponges and the chemical structure of such compounds. The extraction process applied to obtain the compounds will be discussed further, including the parameters that affect the extraction and the types of extraction that can be applied.

### 3.1. Extraction Process

The extraction process is a step that involves the separation of the desired natural products from the raw materials, i.e., the marine sponges [51]. Various methods can be applied to obtain the crude extract [52]. The crude extract is known to be composed of a complex mixture of various metabolites, including alkaloids, terpenoids, peptides and quinones [53,54]. The extraction process can be divided into two categories, which are the traditional extraction method and newly emerging technologies based on the energy or mechanism [52].

#### 3.1.1. Traditional Extraction Method

The traditional extraction method consists primarily of extraction via solvent or solid-liquid extraction [52]. The Soxhlet method involves boiling the sample along with the solvent, with or without stirring, for a certain duration, whereas maceration involves the sample being soaked in the solvent and stirring from time to time [55]. These techniques are examples of solid-liquid extraction methods that can be practiced. These methods have been practiced widely for a long period. However, there are few disadvantages to applying these techniques, which include that they are time-consuming, they require a large volume of solvents, there can be a loss of compounds during the concentration step due to volatilization, there can be hydrolysis of the compounds due to harsh conditions such as high temperature, and solvent wastes can cause environmental pollution [56].

The solvents used in these techniques consist of either a single solvent or a mixture of solvents with a wide range of polarity [57,58]. For example, methanol, ethanol, trichloromethane, acetone and water can be utilized individually as a solvent or they can be utilized with a mixture of organic solvents such as ethanol and acetone [59,60].

#### 3.1.2. Emerging Technology

As there are various limitations to the traditional extraction method, new methods are being practiced that are less time-consuming and that have been found to be more efficient in the extraction process. Microwave-assisted extraction (MAE), ultrasound-assisted extraction (UAE) and pressurized solvent extraction (PSE) are few new techniques that are being widely used as alternative extraction procedures [52,56].

MAE is a technique that utilizes microwave irradiation to speed up the removal of various compounds from the natural matrices [52]. In this method, both heat and mass gradients work in the same direction; that is, friction between the polar molecules results in heat production within the matrix [61,62]. The friction between the molecules is caused by the electric field, resulting in the vibration and or oscillation of the molecules and causing inter- and intra-molecular friction [56]. This results in rapid heating (in seconds) of the matrix, leading to the pressurized effect and causing rupture of the cell wall and membrane. As a consequence, the transfer rate of the compounds from the cells to the solvent occurs at a faster rate [52].

UAE is another technique that utilizes ultrasound waves to alter the physical and chemical properties after interacting with the material [63,64]. The advantages of the technique are that it reduces the extraction duration significantly while simultaneously increasing the extraction yield [65]. This can be achieved due to the formation of cavitation bubbles in the solvent [65]. The cavitation bubble is known to be formed in the liquid during the expansion phase. The ability to form a cavitation bubble depends on the characteristics of the ultrasound wave, the property of the solvents, and the surrounding conditions [66,67]. After a cavitation bubble is formed, it collapses during the compression cycle, resulting in the liquid molecules being pushed together and creating a high-speed micro-jet that moves towards the matrix particle and results in the mixture of the solvent with the matrix [61,66]. In this procedure, the pressure and temperature can reach up to 1000 bar and 4726.85 °C, respectively [66,67]. This results in the breakage of the cell wall and membranes, thus allowing the solvent to penetrate the solid particle easily, releasing the intracellular compounds.

PSE is one of the emerging techniques that utilizes temperature and pressure ranging from 50–200 °C and 35–200 bars, respectively. The temperature and pressure applied in this procedure are set to be lower than the critical temperature (Tc) and critical pressure (Pc) of the solvents being used; hence, the solvents stay in the liquid state [68,69]. The high pressure applied in the procedure results in the rise of the solvent above its boiling temperature; this increase in temperature enhances the solubility and mass transfer rate and simultaneously reduces the viscosity and the surface tension of the solvent [69,70]. The most commonly used solvent in this procedure consists of water, propane and dimethyl ether [60,61,62].

#### 3.1.3. Factors Affecting the Extraction

The extraction process of the bioactive metabolites from marine sponges depends on the type of extraction process that will be applied along parameters such as solvent polarity, temperature and pressure [53,71]. These factors influence the types of metabolites found predominantly in the crude extract. The type of solvents used for extraction can be categorized based on polarity, ranging from non-polar solvents such as hexane and trichloromethane to the highly polar solvent of water [54,72]. A mixture of water and an organic solvent such as ethanol will result in an increase in the yield of phenolic compounds compared to the percentage yield when the solvents are used individually [52].

A study was conducted to identify the influence of the solvents on the percentage yield of the crude extracts by testing the sponges *Stylotella aurantium* (Kelly-Borges and Bergquist, 1988) and *Halicona molitba* (de Laubenfels, 1949) using methanol and water as solvents [73]. The methanol extract of both the sponges exhibited a better antimicrobial activity against *B. cereus* compared to the water extract; however, the water solvent extract of both the sponges exhibited a higher percentage yield. The difference in the yield percentage between the two solvents is due to the polarity. As water is higher in polarity compared to methanol, more polar compounds from the sponges can be extracted, thus increasing the yield percentage [73]. The difference in the antimicrobial activity between the crude extracts suggests that the bioactive compounds that are responsible for the inhibition of the bacterial cell growth are mostly semi-polar; thus the extraction of these compounds was possible with methanol, as it is a semi-polar solvent.

A study was conducted on the marine sponge *Xestospongia* sp. (Laubenfels, 1932) by Bayona et al. (2018) looking at the correlation between the parameter pressure, temperature and solvent polarity and the diversity in the extracted compounds [53]. It was found that the solvent polarity and temperature applied during the extraction process are the most important factors influencing the metabolic diversity in the extract. As mentioned above, an increase in the solvent polarity was found to be positively correlated with the diversity in the compounds extracted. Ethanol was found to be a more suitable solvent, as it resulted in a wide range of compounds compared to the solvent with a mixture of ethanol and dichloromethane. The ethanol solvent was identified to be more proficient in the extraction of polar compounds and also lipophilic fatty acid compounds, thus resulting in a wide range of yield. When the solvent dichloromethane was used, the extract was found to contain compounds belonging to sterols and fatty acids.

However, when the extraction was conducted at a higher temperature (60–80 °C), there was a reduction in the chemical diversity compared to the lower temperature (30–50 °C). The extraction was performed using 100% ethanol to determine the effect of the temperature [53]. The extraction at different temperature ranges resulted in a specific group of compounds being extracted. At the high temperature, specific groups such as aromatic-based compounds were extracted efficiently, whereas at low temperatures, the extracts were identified to contain significant chemical diversity.

Another factor that was reported to significantly influence extraction was the number of cycles used to perform the extraction; this showed an interaction with the temperature at which the extraction process was conducted [53]. The study found that there was no difference in the diversity of compounds obtained when the extraction was performed at low temperature and one or three cycles. However, when the temperature was increased, the diversity in the extract decreased when one cycle was used, though increasing the number of cycles increased the compound diversity.

### 3.2. Classification of Compounds

While the crude extract of known organisms has been identified to contain a wide range of compounds from various chemistry classes, three major classes have been predominantly identified among the marine sponge extracts. A few examples of compounds extracted from sponges will be discussed in this review.

#### 3.2.1. Terpenes

Secondary metabolites consist of terpenes in a large number, where they are synthesized in the form of polymers of isoprene (C_5_H_8_), joined in a repetitive head-to-tail manner [3,74,75]. The modification in the structures of terpene-based metabolites results in a diverse range of derivatives, where there is a wide range of chemical structure and, at the same time, biological properties. Terpenes have an incredible range of structures that can be extracted based on the polarity of the solvent [76]. The terpenes consist of non-polar terpenes, which are linear and cyclized terpenes, and polar terpenoids. The first terpene that was discovered in the marine sponge was steroidal terpenoids. Sesterterpenoid (C25) and triterpenoids (C30) are two major classes of terpenoids that have been commonly isolated from marine sponges and are known to have various bioactivities [42,77].

For example, manoalide is a parent compound of various sponge metabolites belonging to the sesterterpene class and was isolated from sponge *L. variabilis* in Palau [50]. The compound exhibited various bioactivities, such as antibacterial activity against *Streptomyces pyogenes* and *S. aureus*, and is a potent inhibitor of phospholipase A2 (PLA2) enzyme, which is responsible for providing a substrate of pro-inflammatory mediators [78,79]. Furthermore, it was found that the compound manoalide and its analogues’ bioactivities are contributed by the presence of various functional groups in the compounds [3].

Isomalabaricanes are triterpenes that were first identified from sponges *Jaspis stellifera* (Carter, 1879) in Fiji and *Stelletta* sp. (Schmidt, 1862) in Somalia [80,81]. The isomalabaricane triterpenes can be classified into three groups based on the presence of polyene conjugated functionality: (i) stelletins, which possess γ-pyrone; (ii) stelliferins, which can be oxygenated at C-22; (iii) globostellatic acids, which have a carboxyl group at C-4 [3]. Stelletins isolated from marine sponges such as *J. stellifera* and *Stelleta tenuis* (Lindgren, 1897) have exhibited cytotoxic activity against cancer cells such as murine leukemia P388 cell lines [47].

#### 3.2.2. Alkaloids

Alkaloid-based compounds are another class of metabolites that have been extensively identified from marine sponges. The manzamines are polycyclic, β-carboline-derived alkaloids; manzamine A was the first isolated from marine sponge *Haliclona* sp. in 1986. The manzamine compounds are characterized by a fused and bridged tetra- or penta-cyclic ring system linked to the β-carboline [48]. The manzamine alkaloids have various bioactivities, such as antibacterial, anti-malarial and cytotoxic [82,83]. 

Furthermore, bromopyrrole alkaloids contain compounds that can only be found among marine sponges. Oroidin was the first compound isolated from this group, being isolated from *Agelas oroides* (Schmidt, 1864) in 1971. For the compounds classified as bromopyrrole alkaloids, oroidin is the precursor, as these compounds contain a pyrrole-imidazole unit that is a derivative of oroidin [3,45]. Hymenidin, clathrodin and sventrin are examples of bromopyrrole alkaloids that have been isolated from marine sponges, where the bioactivity is attributed to the bromination pattern of the pyrrole moiety [3].

#### 3.2.3. Peptides

Bioactive peptides are a well-established sector of marine natural product-based research, and the peptides from marine sponges have been discovered to contain unique structures compared to the bioactive peptides from different sources. These peptides are cyclic or linear in shape and have amino acids that are either rare or absent in terrestrial-based and microbial peptides [84,85]. Peptides isolated from marine sponges are known to be safe, inexpensive and having a wide range of antimicrobial properties [86].

Antimicrobial peptides (AMPs) are an important source of new antibiotics, which can be used as an alternative to the available antibiotics or in combination with these antibiotics [87]. An example of AMP would be Discodermin A, a tetra-decapeptide; it is the first bioactive peptide isolated from *Discodermia kiiensis* (Hoshino, 1977) from Shikine Island, Japan [46]. Further investigation has resulted in the discovery of various analogues, including discodermin B, C and D [88,89]. Discodermins A–D has exhibited antibacterial activity and is also a potent inhibitor of enzyme PLA2 [3,90]. Tetra-decapeptide AMPs are characterized based on the presence of anomalous amino acid residues, which include formyl-D alanine, 3-methyl-L-proline, and 3-methyl-D-valine [87]. The genus *Theonella* (Gray, 1868) is known as a source of unusual peptides, such as Theonellamide F, which is a bicyclic peptide consisting of a histidino-alanine bridge and exhibiting antifungal properties against various pathogenic fungal strains, such as *Candida* sp., *Trichophyton* sp. and *Aspergillus* sp. [91].

Other examples of peptides are the proline-rich cyclopeptides, which exhibit bioactivities such as anti-cancer, immunosuppressor, and as anti-inflammatory [87]. The proline residue found in the peptide molecules is important for structural stability by reducing the conformational flexibility. This is due to the rigidity provided by the proline ring, leading to the maintenance of the rigid structure of the peptide [65]. Examples of proline-rich cyclopeptides are Callyaerins A–F and H, which are extracted from ethyl acetate extract of marine sponge *Callyspongia aerizusa* (Desqueyroux-Faùndez, 1984) [87]. One of the compounds, Callyaerin A, showed potent antifungal activity against *Candida albicans*.

## 4. Bioactivities of Marine Sponges With Regard to Human Diseases

In recent years, the research community has been focused on finding alternatives to overcome increasing cases of antimicrobial-resistant microorganisms such as bacteria and fungus [75,92]. Natural products from plants and microorganisms are seen as an alternative to overcome antimicrobial resistance [93]. Similarly, marine organisms—especially sponges—have been reported to synthesize bioactive compounds that have antimicrobial activity. For example, the marine species belonging to the genus *Erylus* (Gray, 1867) from the Mediterranean Sea and the Atlantic and Pacific Oceans are found to be able to synthesize glycolipids, which are capable of inhibiting the growth of bacteria, fungi and viruses, including HIV [94]. Table 3 summarizes various antimicrobial activities from different marine sponges. In the following subsections, the antimicrobial activities of marine sponge bioactive compounds will be discussed.

### 4.1. Antibacterial Activity 

In general, bacteria can be categorized based on the cell wall structure, which is Gram-positive or Gram-negative [105,106]. Gram-positive bacterial cell walls comprise a thick peptidoglycan layer containing teichoic acid, lipoteichoic acid and proteins embedded within the peptidoglycan layer, whereas Gram-negative bacteria have a thinner peptidoglycan layer surrounded by an outer membrane layer containing lipopolysaccharides [75,107]. Despite the differences in structure, there are various compounds isolated from marine sponges that have been shown to exhibit antibacterial properties against pathogenic human bacterial strains such as *S. aureus*, *Eschrichia coli* and *Pseudomonas aeruginosa*. In general, bioactive compounds may cause death through the leakage of the contents in the bacterial cells due to disruption of the cell membrane through the generation of reactive oxygen species (ROS). ROS is also able to oxidize the proteins found within the bacterial cells by altering the covalent bonds that are responsible for maintaining the structure of the proteins essential for the survival of the cells [108,109].

Clathric acid, a bicyclic terpenoid, was isolated from *Clathria compressa* collected from Florida, USA. It exhibited antibacterial activity when tested against methicillin-resistant *S. aureus* ATCC 33,591 (MRSA), *S. aureus* ATCC 6538P and vancomycin-resistant *S. aureus* (VRSA) bacterial strains with MIC values of 64 µg/mL, 32 µg/mL and 64 µg/mL, respectively [96].

Lectin is a glycoprotein compound found in *Axinella donnani*. It showed high potency in anti-biofilm activity against biofilm-producing *S. aureus* and at the same time showed antibacterial activity against *S. aureus* bacterial cells [95]. The total biomass of the biofilm was reduced in a concentration-dependent manner after being treated with lectin at a concentration of 15.1 µg/mL to a concentration as high as 1000 µg/mL; the treatment at 1000 µg/mL showcased an inhibition of more than 80%.

However, some of the marine sponge compounds were found to be less potent in Gram-negative bacterial strains. For example, clathric acid isolated from *C. compressa* did not exhibit any antibacterial activity at a concentration of 128 µg/mL when tested against *Escherichia coli* and *Klebsiella pneumoniae*; however, as stated above, the compound exhibited antibacterial activity against Gram-positive bacteria [96]. The extracts that were found to exhibit antibacterial activity against Gram-negative bacteria are the compound lectins extracted from *A. donnani*, which simultaneously exhibited anti-biofilm activity against Gram-negative bacterial strains *E. coli, K. pneumoniae* and *Pseudomonas aeruginosa* and potent anti-biofilm activity against biofilm-producing *P. aeruginosa* [95]. Similarly, extract from *Haliclona* also exhibited antibacterial activity against Gram-negative bacterial strains *E. coli* and *P. aeruginosa*, with the same potency as the Gram-positive bacterial strains [110].

The compound nagahamide A is a depsipeptide that is isolated from *Theonella swinhoei* (Gray, 1868). The compound was found to exhibit a weak antibacterial activity against *E. coli* and *S. aureus* [111]. Nagelamides Q and R are dimeric bromopyrrole alkaloids isolated from *Agelas* sp.; they were found to exhibit antibacterial activity. The compound nagelamide Q was found to contain a pyrrolidine ring, whereas nagelamide R would be the first bromopyrrole alkaloid containing an oxazoline ring [112]. Both the compounds exhibited significant inhibition against *B. subtilis,* with an MIC value of 13.0 µg/mL, whereas both the compounds exhibited activity against *E. coli, M. luteus* and *S. aureus* at concentrations greater than 25 µg/mL.

Compounds such as a bisindole alkaloid 2,2-bis(6-bromo-3-indolyl)ethylamine and its synthetic analogues were able to exhibit antibacterial activity and simultaneously inhibit the formation of biofilm. The compound 2,2-bis(6-bromo-3-indolyl)ethylamine, isolated from the New Calodenian sponge *Orin asp*. (Gray, 1867), exhibited significant antibacterial activity against *E. coli¸ S. aureus* and *K. pneumoniae* with an MIC concentration as low as 8 mg/L. The compound was also found to be able to inhibit the biofilm formation simultaneously by 82.2% and disaggregate the biofilms of the *E. coli, S. aureus,* and *K. pneumoniae* pathogens [113].

### 4.2. Antiviral Activity

Viruses are obligate intracellular parasites that are small in size and contain a genome, either RNA or DNA surrounded by a protective layer known as a virus-coded protein coat [114]. Over the centuries, viruses have been responsible for various diseases, such as chicken pox, AIDS, SARS and measles as well as the current pandemic causative agent for severe acute respiratory syndrome coronavirus 2 (SARS-CoV-2) [115]. Various bioactive compounds extracted from marine sponges showed antiviral effects against herpes simplex virus and Hepatitis A virus. 

Compounds stachybogrisephenone B, grisephenone A, and 3,6,8-trihydroxy-1-methylxanthone are compounds that were isolated from a fungal strain, *Stachybotry* sp. HH1 ZSDS1F1-2, which was isolated from a marine sponge from Xisha Island, China [100]. The compounds exhibited antiviral activity against the intestinal virus Enterovirus 71 (EV71), where the compounds each exhibited IC_50_ (concentration that can inhibit the virus activity by 50%) values of 30.1 µM, 50 µM, and 40.3 µM compared to the positive control ribavirin, which had an IC_50_ value of 0.60 µM. All the compounds that exhibited antiviral activity are xanthone derivatives.

Another compound that exhibited antiviral activity is metachromin A, a sesquiterpene compound isolated from *D. metachromia* [99]. The compound was able to inhibit the Hepatitis B viral production significantly at an EC_50_ (concentration where 50% of target cells are protected from death due to infection by the virus) value of 0.8 µM and SI (selectivity index) value of 19.6. The antiviral activity of the compound is contributed by the presence of hydroquinone moiety and the presence of double bond at C-5 and C-9 of the compound, which are attributed to the inhibition of the core promoter of the Hepatitis B viral cells and production of the viral particles. The compound also was identified to suppress the viral replication via down-regulation of the promoter activity.

Another two compounds that were found to exhibit antiviral activity against Hepatitis B viral cells were 3,5-dibromo-2-(2,4-dibromophenoxy)-phenol and 3,4,5-tribromo-2-(2,4-dibromophenoxy)-phenol, which belong to the polybrominated diphenyl ethers, isolated from *Dysidea* sp. [116]. Both of these compounds were able to inhibit the production of Hepatitis B viral cells in the cell line HepG2.2.15.7 in a dose-dependent manner, with an EC_50_ value of 0.23 µM for 3,5-dibromo-2-(2,4-dibromophenoxy)-phenol and 0.80 µM for 3,4,5-tribromo-2-(2,4-dibromophenoxy)-phenol and SI values of 18.2 and 12.8, respectively. However, both the compounds were found to be more toxic compared to the control entecavir. 

Other than Hepatitis B virus, the compound manoalide, which was first isolated from marine sponge *L. variabilis*, exhibited antiviral activity against Hepatitis C virus [117]. The compound was found to inhibit the RNA helicase and ATPase activities of the NS3 protein of Hepatitis C virus in a dose-dependent manner, with IC_50_ concentrations of 15 µM and 70 µM, respectively. The compound was found to act as a non-competitive inhibitor by inhibiting the NS3 protein from binding to the single-stranded RNA. NS3 is a non-structural protein that involves the coordination of the intracellular process of the Hepatitis C viral particle [118]. Another compound that exhibited antiviral activity against Hepatitis C virus is the compound psammaplin A, a biphenolic bromotyrosine-derived compound [119]. The compound is commonly isolated from marine sponge *Psammaplysilla* (Keller, 1889) sp., *Poecillastra* (Sollas, 1888) sp. and *Jaspis* (Gray, 1867) sp. Similar to the compound manoalide, psammaplin A also exhibits antiviral activity by inhibiting the NS3 protein from binding to the ssRNA in a dose-dependent manner. The compound acts as a non-competitive inhibitor on the helicase motif by binding to the allosteric site, thus modulating ATPase and RNA binding activity via conformational changes of the helicase. The compound also was able to inhibit the viral replication of Hepatitis C at EC_50_ values of 6.1 and 6.3 µM in subgenomic replicon cells [119]. Thus, the compound, similar to manoalide, exhibited the ability to inhibit ATPase, RNA binding and helicase activity of NS3.

Herpes simplex virus type-1 (HSV-1) is a causative agent of skin infection that results in mucocutaneous lesions known as herpes labialis. The compound manzamine A, a β-carboline alkaloid, which is commonly found in marine sponges genus *Haliclona*, *Chalinidae* (Gray, 1867), *Niphatidae* (van Soest, 1980), *Petrosidae*(Boury-Esnault and van Beveren, 1982), *Thorectidae* (Bergquist, 1978) and *Irciniidae* (Gray, 1867) family [120,121]. The compound manzamine A, which was extracted from marine sponge genus *Acanthostrongylophora* (Hooper, 1984), exhibited antiviral activity against HSV-1 by inhibiting the replication of the viral particle in a corneal cell line (SIRC) at a concentration as low as 1 µM and an IC_50_ value of 5.6 µM [122]. Manzamine A at the concentration of 1 µM was shown to be able to reduce the plaque formation by 1011-fold. The compound was found to result in down-regulation of the gene ICP0, which is an infectious cellular protein 0 that is produced in the early stage of infection after entering the host cells.

Various compounds isolated from marine sponges have exhibited varying degrees of potency towards HIV. The compounds baculiferins C, E–H, and K–N, amino acid DOPA (2-amino-3-(3′,4′-dihydroxypenyl) propionic acid)-derived alkaloids from marine sponge *Iotrochota baculifera* (Ridley, 1884), and the compounds stellettapeptins A and B, depsipeptide compounds isolated from marine sponge *Stelletta* sp. (Schmidt, 1862), have exhibited potent activity against HIV viral particles [123,124]. The compounds baculiferins C, E–H and K–N were potent against the cell lines MT4 and MAGI cells encoded with HIV-1 IIIB virus. The IC_50_ values of these compounds for both the cell lines range from 0.1 µg/mL to 8.4 µg/mL [123]. The compounds stellettapeptines A and B showcased EC_50_ values of 23 nM and 27 nM, respectively, against the human T-cell line CEM-SS, infected with HIV-1RF [124].

### 4.3. Antifungal Activity

*C. albicans* is the fungal species that is the frequent causative agent of candidiasis. It has been estimated that human fungal diseases result in the death of more than 1.6 million people every year [125]. Hence, it is necessary to discover new antifungal compounds to ensure these diseases can be treated. There are few compounds extracted from marine sponges that are known to exhibit antifungal activity [126].

Xestodecalactones A, B and C are compounds that have been isolated from the marine sponge *Xestospongia exigua* from Bali Sea, Indonesia [102]. The compound was synthesized by the fungus *Penicillium* cf. *montanense*, which formed a symbiotic interaction with the marine sponge. The compound xestodecalactone B was able to inhibit the growth of *C. albicans* at concentrations of 20 µM or higher. 

Another example of compounds with antifungal activity is nortetillapyrone, a tetrahydrofurylhydroxypyran-2-one from the marine sponge *Haliclona cymaeformis* (Esper, 1806). It exhibited antifungal activity against various fungal strains [127,128]. The compound exhibited moderate activity against fungal strains *C. tropicalis* with an MIC value of 250 µg/mL, good activity against *C. glabata, Microsporum camis,* and *C. neoformans* at an MIC of 31.25 µg/mL and against *C. dubliniensis*, and *Trichophyton rubrum* at an MIC value of 62.5 µg/mL. The compound woodylides A and C, a linear polyketide from marine sponge *Plakortis simplex* (Schulze, 1880), was found to exhibit moderate antifungal activity against the fungal strain *C. neoformans*, with IC_50_ concentrations of 3.67 µg/mL and 10.85 µg/mL, respectively [129]. Both the compounds are categorized as acyclic diol analogues of cyclic polyketide peroxides. 

The compounds theonellamide G, a bicyclic glycopeptide, and swinholide I and hurghadolide A macrolides, are isolated from marine sponge *T. swinhoei.* The compounds were found to exhibit significant antifungal activity against the fungal strain *C. albicans* wild strain, an amphotericin-B resistant strain where the compound theonellamide G showcased an activity with IC_50_ values of 4.49 µM and 2.0 µM, respectively. The compound swinholide I showcased moderate inhibitory activity against *C. albicans* wild strain with an MIC of 62.2 µg/mL, whereas amphotericin-B resistant strain exhibited activity at 500 µg/mL, and the compound hurghadolide A exhibited potent activity against both the strains with an MIC value of 31.3 µg/mL [130,131]. 

Other compounds extracted from *T. swinhoei* that have exhibited antifungal activity are aurantosides G and I, which are tetramic acid glycosides. The compound aurantoside G exhibited poor activity against *Fusarium solani* with an MIC concentration of 16 µg/mL but moderate activity against the fungal strains *C. albican,* and *C. glabrata* with an MIC concentration of 4 µg/mL and against *C. parapsiolsis* and *C. tropicalis* with an MIC concentration of 2 µg/mL [132]. The compound aurantoside I exhibited significant antifungal activity against *C. albicans* and *C. tropicalis* at 0.25 µg/mL; *C. parapsiolsis* at 0.50 µg/mL; *C. glabrata* with an MIC concentration of 0.125 µg/mL; and *F. solani* with an MIC concentration of 1 µg/mL [132].

### 4.4. Antiparasite Activity

It is interesting to note that marine sponge compounds also display good anti-malarial activity. Meroterpenes, alisiaquinone A, alisiaquinone B, alisiaquinone C and alisiaquinol isolated from New Caledonian deep-sea sponge aqueous ethanol extract have been shown to exhibit anti-malarial activity against enzymes plasmodium kinase Pfnek-1 and protein farnesyltransferase, which can be used as inhibitors to treat malaria [103]. These compounds also exhibited anti-malarial activity against chloroquinone-resistant *Plasmodium falciparum* strain, which demonstrated the potential of the usage of these compounds in malarial resistance. Similarly, sesquiterpenoid compounds isolated from marine sponge *Hyrtios erectus* (Keller, 1889) from Chuuk Island, Federated States of Micronesia, exhibited anti-malarial activity against chloroquinone-resistant Dd2 strain *P. falciparum* [133]. The compound pelorol exhibited strong anti-malarial activity at a concentration of 0.80 µM against *P. falciparum*, whereas compounds smenotronic acid and limaquinone exhibited moderate activity against the strain at 3.51 µM and 2.11 µM, respectively.

Marine sponge compounds have been identified to have antiparasitic activity against parasites *Leishmania*, which causes Leishmaniasis, and *T. cruzi*, which causes Chagas disease, along with anti-malarial activity. Three compounds extracted from marine sponge *V. rigida*—11-hydroxyaerothionin, purealidin B and aeroplysinin-A—exhibited leishmanicidal activity, anti-malarial activity and anti-chagas activity, respectively [104]. These compounds are bromotyrosine compounds, and the various bioactivities of these compounds are due to the presence of a cyanide group that is very reactive. The cyanide group present in the compound acts as an inhibitor of cytochrome C oxidase enzyme, preventing electron transports that produce ATP, resulting in cell apoptosis [104].

## 5. Applications in Aquaculture

In recent years, the aquaculture industry has rapidly increased globally. The major limitation in the aquaculture industry is the occurrence and spread of diseases in fishes; this affects the production rate, incurring a huge financial loss [134]. It has been estimated that the aquaculture industry faces a total loss of more than US $6 billion per year due to frequent outbreaks of diseases primarily caused by bacterial, viral, fungal and/or parasitic attacks [135]. The most common diseases of freshwater and marine fish are *Aeromonas* sp., *Vibrio* sp. and *Streptococcus* sp. [136,137]. The discovery of bioactive compounds from marine sponges and their use as drugs may help in disease management in the aquaculture industry [138]. 

Marine sponges such as *A. donnani, Acanthella elongata* (Dendy, 1905)*, Echinodictyum gorgonoides* (Dendy, 1916)*, Callyspongia subarmigera* (Ridley, 1884) and *C. diffusa* (Ridley, 1884) have been reported to be highly effective in antibacterial activity. The compounds isolated from crude extracts of marine sponges were capable of controlling the pathogens of fish and shrimp, especially *Aeromonas hydrophila, P. aeruginosa, V. alginolyticus, V. fischeri, V. vulnificus, V. pelagius, V. fluvialis* and *V. anguillarum* [138].

Symbionts such as bacteria and archaea synthesize bioactive compounds, which are utilized by the sponges as natural defense mechanisms for survival [139]. For example, the *Bacillus* strains isolated from marine sponges *Hyrtios* sp., *Verongula* sp. and *Smenospongia* sp. have antibacterial activity against fish pathogens that cause vibriosis, such as *V. harveyi*, *V. parahaemolyticus* and *V. vulnificus* [140]. 

Sponges are rich in various chemical derivatives, mimicking potent natural biocides in the development of natural product antifoulants (NPAs). The rapid expansion of the aquaculture industry requires various drugs, especially antifoulants, to inhibit the growth of unwanted microorganisms (such as barnacles) in aquaculture facilities. A study conducted on the marine sponge *Topsentia ophiraphidites* (Laubenfels, 1934) reported that the extracts of this sponge have chemical properties that could be used as bioactive components in antifouling paint [141]. Table 4 lists several compounds extracted from marine sponges that are beneficial to the aquaculture industry. 

### 5.1. Antimicrobial Activity Against Fish Pathogens

Compounds from marine sponges have shown to exhibit various antimicrobial activities, such as antibacterial, antiviral and antifungal properties against various fish pathogens, similar to human pathogens.

#### 5.1.1. Antibacterial

An example of a compound that has exhibited antibacterial activity against fish pathogens is long-chain fatty acid 9,12-Octadecadienoic acid, which was extracted from marine sponge *C. diffusa* (Ridley, 1884) from the Southwest coast of India [143]. The compound was isolated from the methanol extract of the sponge, and 30 µL of the extract was tested for potency against various fish pathogens. The extract exhibited moderate antibacterial activity against fish pathogens *V. fluvialis, V. harveyi* and *V. vulnificus* by exhibiting 8 mm, 7 mm and 8 mm zones of inhibition (diameter), respectively. The compound showcased potent antibacterial activity against *V. anguillaram*, with a 12 mm zone of inhibition [143].

Other examples of compounds that exhibited antibacterial activity are the compound sesquiterpene axisonitrile-3, isolated from sponge *A. kletra* (Pulitzer-Finali, 1982), and diterpene isonitrile, isolated from marine sponge *C. hooperi* van Soest (Desqueyroux-Faùndez, Wright and König, 1996) [143]. Both compounds exhibited significant antibacterial activity against *V. harveyi*; the compound diterpene isonitrile was significantly potent at concentration 2.5 µg/mL, whereas axisonitrile-3 activity was observed at the concentration 250 µg/mL.

*S. tirandamycinicus* is an actinobacterium that co-exists with a marine sponge located on the coast of Wenchang City, Hainan Province, China. It synthesizes compounds tirandamycin A and B [145]. Both the compounds showcased potent inhibitory activity against *S. agalactiae* HNe0 strain, with MIC values of 2.52 and 2.55 µg/mL, respectively. The compounds contain a bicyclic ketal and dienoyl tetramic acid moiety. The compounds were found to be able to inhibit RNA polymerase of bacteria and also found to be a specific inhibitor of the futalosine pathway, which is responsible for the synthesis of menaquinone, a crucial component in the electron-transfer system of prokaryotes [153,154].

Three bromotyrosine compounds 11-*N*-methylmoloka’iamine, 11-*N*-cyano-11-*N*-methylmoloka’iamine, and kuchinoenamine are isolated from marine sponge *Hexadella* sp. [144]. The compounds were found to exhibit moderate antibacterial activity against *A. hydrophila*, a fish pathogen, at a concentration of 100 µg/diameter 6.5 mm disk. The compounds 11-*N*-methylmoloka’iamine, 11-*N*-cyano-11-*N*-methylmoloka’iamine and kuchinoenamine showcased zones of inhibition with a diameter of 7.5 mm, 8 mm and 7 mm, respectively.

Poly-β-hydroxybutyric acid (PHB), a polymer synthesized by bacteria *Brevibacterium casei* MS104, found to co-exist on the marine sponge *Dendrilla nigra* (Dendy, 1889), was found to exhibit anti-biofilm activity [155]. The compound was found to be able to inhibit the formation of biofilm against shrimp pathogens such as *V. harveyi, V. alginolyticus, V. vulnificus, V. fischeri* and *V. parahaemolyticus* at a concentration of 600 µg. The compound effectively reduced anti-adhesive activity by 96% against *V. vulnificus* and *V. fischeri,* followed by 92% inhibition for *V. parahaemolyticus* and *V. alginolyticus*, and 88% inhibition against *V. harveyi*. The compound PHB is one of the derivatives of the polymer poly-hydroxy alkanoates.

*Aspergillus* sp. LS116 strain, which is found on the marine sponge *Haliclona* sp., was discovered to synthesize the compounds aspergillsteroid A and neocyclocitrinol B, both belonging to steroids with bicyclo A/B rings in their structure [156]. The compound aspergillsteroid A was found to have potent antibacterial activity against *V. harveyi* KP635244 strain at a concentration of 128 µg/mL, with the growth of the bacteria inhibited, whereas the compound neocyclocitrinol B showcased a weak activity at the same concentration against the bacterial strain. Despite belonging to the same group of classification, the difference in the antibacterial activity between the two compounds is attributed to the absence of C-23 hydroxyl branch moiety in the compound aspergillsteroid A [156]. 

#### 5.1.2. Antifungal and Antiviral

The compound latrunculin B was isolated from the marine sponge *N. magnifica* (Keller, 1889) from Hurghada, Red Sea; it exhibited antifungal activity against fish fungal pathogens *E. salmonis, B. demigrans* and *Saprolegnia* sp. [146]. The compound latrunculin B structure was identified to contain macrolide 1,3, which are fused with a tetrahydro-puran, which contains a side chain 2-thiazolidinone [146]. The compound exhibited potent antifungal properties against *E. salmonis, B. demigrans* and *Saprolegnia* sp. by having very low MIC values of 1.50 µg/mL, 0.75 µg/mL and 3.0 µg/mL, respectively. The compound was also identified to be non-toxic towards shrimp [146,157].

An example of a viral diseases that affects the aquaculture industry of shrimp farming is white spot syndrome virus (WSSV), which causes 100% mortality among infected shrimps within 2 to 10 days [158]. The compound polyhydroxy isocopalane, a terpene, was extracted from marine sponge *Callyspongia* sp. in Indonesia [147]. The compound was found to exhibit strong antiviral activity against the WSSV at a concentration of 60 mg/L and resulted in 34% survival of the WSSV infected *Litopenaeus vannamei*. The presence of –OH group in the terpene was found to be responsible for inhibiting the replication of the virus in the host organism cell [147].

### 5.2. Antifouling Activity

Biofouling is one of the major issues affecting the aquaculture industry. It is the settling and development of unwanted aquatic species such as plants, microorganisms or small animals on the surface, resulting in plagues in various aquaculture organisms, such as shellfish and seaweed [159]. The costs associated with management of biofouling in the aquaculture industry are estimated to be 5–10% of production costs, and the approaches to the management of biofouling vary between location, species, and company [159].

Marine sponges have proved to contain various compounds that exhibit antifouling activities. One such example is the compound barettin (cyclo[(6-bromo-8-entryptophan)arginine]) as well as 8,9-dihydrobarettin (cyclo[(6-bromotryptophan)arginine]) from marine sponge *Geodia barretti* (Bowerbank, 1858) [160]. Both these compounds, isolated from *G. barretti,* belong to the brominated cyclopeptide class. Both compounds have exhibited the ability to inhibit the cyprid larvae of barnacle *B. improvises*; however, the activity was found to be reversible. The compounds barettin and 8,9-dihydrobarettin were able to inhibit the settlement of the larvae completely at concentrations of 1.9 µM and 19 µM, respectively, in a dose-dependent manner. However, the inhibition by both compounds was found to be reversible when the larvae were transferred from the water with the compounds to freshwater. Both the compounds contain tryptophan, which results in the hydrophobic element, which enables penetration of the compounds into the cell wall and tissues of the larvae [160].

Similarly, the compound agelasine D, extracted from marine sponges of genus *Agelas* (Duchassaing and Michelotti, 1864), also exhibited an inhibitory effect on the settlement of the larvae *B. improvisus* [149]. The compound agelasine D is a 7,9-dialkylpurinium salt with a diterpenoid as a side chain; it inhibited the settlement of the larvae in a dose-dependent manner. The compound inhibited the settlement of the larvae ranging in concentration from 0.024 µM to 24 µM; at concentration 0.24 µM, significant inhibition was observed. However, the compound did not result in the mortality of the larvae at the tested concentration of the compound. The compound agelasine D, from marine sponge *Agelas nakamurai* (Hoshino, 1985), also exhibited a similar result, inhibiting the settlement of *B. improvisus* larvae [161].

The diterpenoid compounds kalihinols A, E, O–T, 10-*epi-*kalihinol X,I, 10β-formamidokalihinool-A, and 10β-formamido-5β-isothiocyanatokalihinool-A, which were isolated from *A. cavernosa* (Dendy, 1922), exhibited significant antifouling activity against the larvae of barnacle *B. amphitrite* at an EC_50_ (concentration that inhibits 50% of the larvae settlement in comparison with the control) of 1.85, 0.92, 1.43, 0.72, 1.48, 1.16, 0.53, 0.74, 0.69, 0.37, 1.37 and 0.41 µM, respectively [148]. These compounds are identified to contain a trans-decalin ring carrying a tetrahydrofuran or a tetrahydropyron ring. 

Compounds diterpene isonitrile, isolated from marine sponge *C. hooperi,* and axisonitrile-3 from marine sponge *A. kletra* showcased the ability to inhibit the settlement of the diatom *Nitzschia closterium* at concentrations of 10 and 100 µg/mL, respectively [142]. The study found that the compound axisonitrile-3 was more significantly potent in reducing the settlement of the diatoms. Marine sponge *D. avara* synthesizes compounds such as avarol, a sesquiterpene hydroquinone, and compound avarone, an oxidized avarol quinone that is able to inhibit the settlement of the *B. amphitrite* at both the nauplii and cyprid stage [162]. Both the compounds avarone and avarol were able to inhibit the settlement of the cyprid and were also found to be toxic, resulting to 50% mortality compared to the control at concentrations as low as 13.28 µg/mL and 27.12 µg/mL, respectively. The compound toxicity (resulting mortality) towards the nauplii of the *B. amphitrite* was found to be lower for both the compounds: 1.58 µg/mL for avarol and 25.12 µg/mL for avarone [162].

Three sesterterpene compounds, *7E,12E,20Z*-variabilin, cavernosolide, and lintenolide A, were extracted from marine sponge *Semitaspongia bactriana* (Cook and Bergquist, 2000) and found to have antifouling activity against the diatom *N. closterium* and bryozoan *Bugula neritina* [163]. The compound *7E,12E,20Z*-variabilin was found to be the most toxic towards the diatom *N. closterium*, with an EC_50_ concentration of 3.52 µM, whereas the compounds cavernosolide and lintenolide-A were less potent against the diatom with EC_50_ values of 5.24 µM and 6.72 µM, respectively. The compounds cavernosolide and lintenolide-A were found to be more potent compared to the *7E,12E,20Z*-variabilin towards the *B. neritina*, with EC_50_ values of 1.22 µM, 1.59 µM and 7.41 µM, respectively [163]. The cytooxic property of the compound *7E,12E,20Z*-variabilin is attributed to the presence of tetronic acid moiety, whereas for the compounds cavernosolide and lintenolide-A, the presence of γ-hydroxybutenolide moiety contributes to the antifouling activity.

Other examples of compounds that exhibited antifouling activity against *B. amphitrite* cyprids are compounds ircinin I & II mixture and ircinin I and II mixture acetates from the sponge *Ircinia oros* (Schmidt, 1864); hydroquinone-A acetate and hydroquinone-C acetate from sponge *Ircinia spinosula* (Schmidt, 1862); furodysinin, euryfuran and 7-deacetoxyolepupuane from *Dysidea* sp.; and compound dihydrofurospongin-II from sponge *Cacospongia scalaris* (Schmidt, 1862) [164]. The compounds ircinin I & II mixture, ircinin I and II acetates mixture, furodysinin and 7-deacetoxyolepupuane were found to be able to inhibit the settlement of the *B. amphitrite*; however, the compounds were found to be toxic, with an LC_50_ (concentration of compounds resulting in 50% mortality) value of 4.7 ppm, 4.9 ppm, 18.1 ppm and 106.2 ppm, respectively. On the other hand, the compounds euryfuran, hydroquinone-A acetate, dihydrofurospongin II and hydroquinone-C acetate were found to be non-toxic and were able to prevent the settlement of the cyprids in a dose-dependent manner. The compounds euryfuran, hydroquinone-A acetate and dihydroquinone-C acetate at a concentration of 100 µg/mL reduced the settlement of the cyprids to 24.7%, 19.9% and 11.2%, respectively. The presence of furan ring in these compounds was found to have exhibited strong settlement inhibition [164].

## 6. Applications in Veterinary Commodities

The benefits of the sponges’ secondary metabolites are not only useful in aquaculture but also have been extended for use in other veterinary commodities to treat various diseases affecting animals, which will be discussed in this section. Table 5 summarizes the application of the marine sponge compounds among veterinary commodities. A few applications from the marine sponge compounds will be discussed in this section.

NOR-Batzelladine L, an extract isolated from the marine sponge *Monanchora* sp. has been shown to have antiviral activity against the avian metapneumovirus (aMPV) [168,169]. The compound was able to inhibit the virus by 97% during the viral adsorption phase. The compound Batzelladine L is a guanidine derived alkaloid. aMPV is a non-segmented single-stranded RNA virus from the family *Paramoxyviridae* [172]. The virus is highly virulent to avian species such as chickens and turkeys and causes a huge loss to the poultry industry [173]. The virus causes rhinotracheitis among turkeys and causes swollen head syndrome among chickens [173]. The illness rate caused by aMPV among chicken flocks ranges between 30–80%, with a mortality rate among these chickens of approximately 15% [172].

The cattle farming industry faces huge economic losses (e.g., dairy milk is always discarded due to infection) yearly to a disease known as mastitis, caused by Coagulase-negative *Staphylococci* (CNS) such as *S. chromogens*, *S. simulans*, *S. hyicus* and *S. epidermidis* [174]. The extracts of marine sponges from Southern Brazil have been reported to harbor potent antibacterial activity against CNS [167]. Both aqueous and ethanolic crude extracts were obtained from the marine sponges *Cinachyrella* sp., *Haliclona* sp. and *Petromica citrine,* and both crude extracts showcased antibacterial activity. Other studies have shown that about 61.2% of bacterial strains from bovine mastitis were inhibited by at least one extract of marine sponges, such as *Cliona celata* complex, *Dragmacidon reliculatum*, *Geodia corticostylifera*, *Guitarra sepia*, *Haliclona* sp. and *Hymeniacidon heliophila*. 

Feline leukemia virus (FelV) is the most common infectious disease in domestic cats and has been reported to have a direct association with anemia, immunodeficiency, leukemia and lymphoma [175]. The virus may affect the organs and may also cause secondary and/or opportunistic infections [176]. The virus belongs to the family Retroviridae, and the pathogenicity is often associated with the insertion of its double-stranded copy of DNA from the single-stranded RNA into the chromosome of the host cells [177]. Once the viral particle enters the host cells and successfully inserts its genome into the host genome, the viral genes are then transcribed into functional protein products. It may also remain latent for some time [177]. An extract isolated from marine sponge *Aplysinu thiona* from the Bahamas was tested against FelV; the extract showed antiviral properties against the viral particles [165]. Fistularin 3 and 11-ketofistularin 3 compounds, which are a derivation of bromotyrosine in the extract, are found to be responsible for the antiviral activity. 

## 7. New Compounds from Marine Sponges and Associates

In this subsection, we will discuss a few compounds that have been recently identified (from 2018–2021) based on various bioactivities. Table 6 below lists the compounds that have been identified from the marine sponges, their bioactivities and the classification of the compounds.

**Figure 4:**
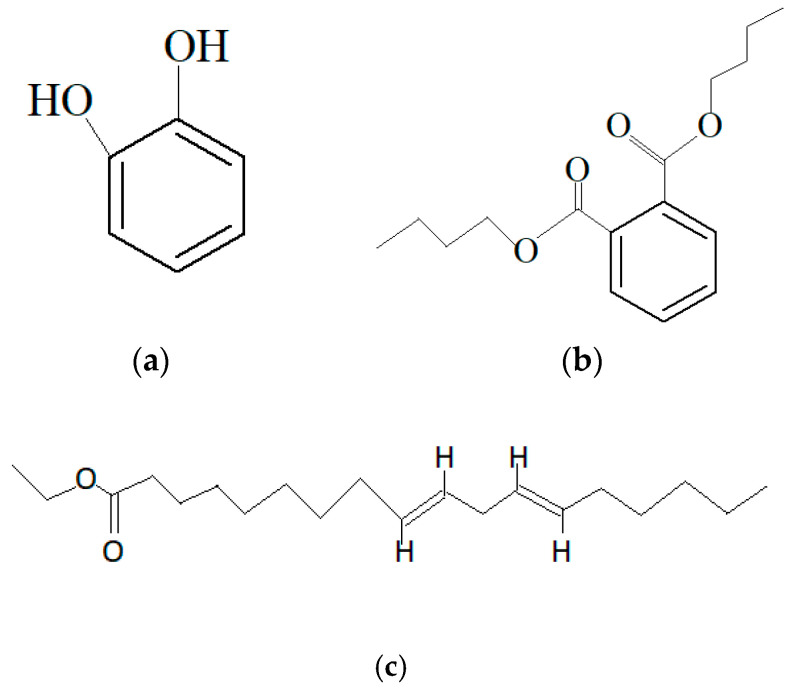
Chemical structures of (**a**) 1,2-Benzenediol; (**b**) Dibutyl phthalate; (**c**) 9,12-Octadecadienoic acid ethyl ester.

*S. carteri* ethanol extract from Indonesia exhibited anti-cancer activity against breast cancer MDA MB 231 cell lines [190]. From the study, it was identified that the extract contains a mixture of compounds: the major compounds that were present in the extract were 1,2-Benzenediol (Figure 4a), Dibutyl phthalate (Figure 4b) and 9,12-Octadecadienoic acid ethyl ester (Figure 4c). Dibutyl phthalate is generally known as a pollutant and is widely used as a plasticizer in the plastic manufacturing industry. However, the presence of the compound in the extract can be due to the synthesis by the microorganism symbionts of the marine sponge. It was found that the compound dibutyl phthalate is synthesized as secondary metabolites by *Pseudomonas* sp. PB01, *Streptomyces ruber* EKH2 and *Rheinheimera japonica* KMM9513, which are marine bacteria species, and filamentous fungi such as *Penicillium lanosum*PTN121, *Trichoderma asperellum* PTN7 and *Aspergillus niger* PTN42 [191,192,193,194]. The extract resulted in the death of the breast cancer cell lines via a dose-dependent manner through apoptosis and also by preventing the migration of the cancerous cells. Dibutyl phthalate has been reported to induce proliferation and invasiveness of breast cancer cells [195].

**Figure 5:**
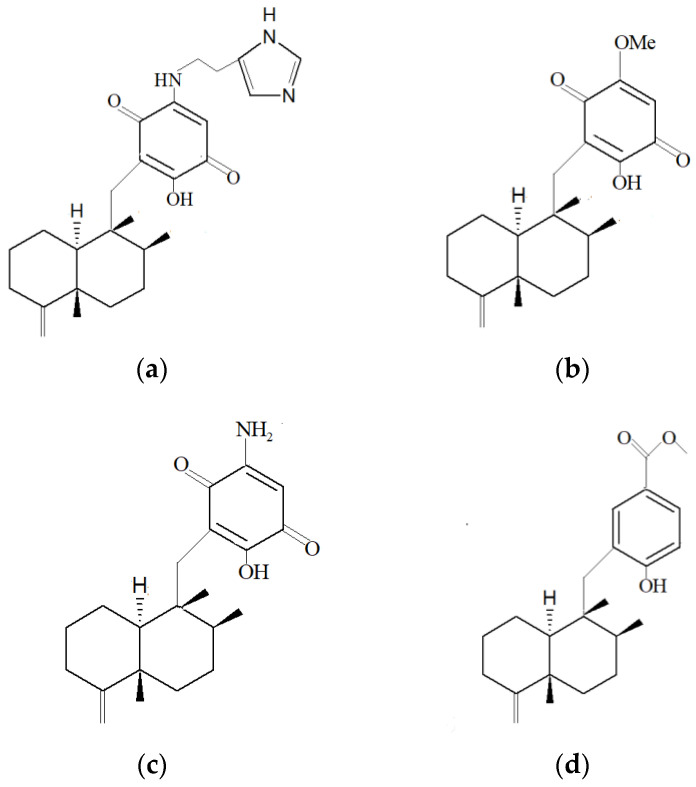
Chemical structures of (**a**) Nakijiquinone V; (**b**) Ilimaquinone; (**c**) Smenospongine; and (**d**) Dyctioceratine C.

In another marine sponge *D. elegans,* T3 methanol extract from Beach Tahuna Bay, North Sulawesi Province Indonesia, exhibited antibacterial activity towards Gram-positive bacteria *Micrococcus luteus* ATCC 4698 and *Bacillus megaterium* DSM32 [181]. The compounds that exhibited antibacterial activity were identified to be sesquiterpene aminoquinone (nakijiquinone V) (Figure 5a), two sesquiterpene quinones (illimaquinone (Figure 5b), and smenospongine (Figure 5c)) and sesquiterpene hydroquinone (dyctioceratine C) (Figure 5d). The compound nakijiquinone was newly discovered.

**Figure 6:**
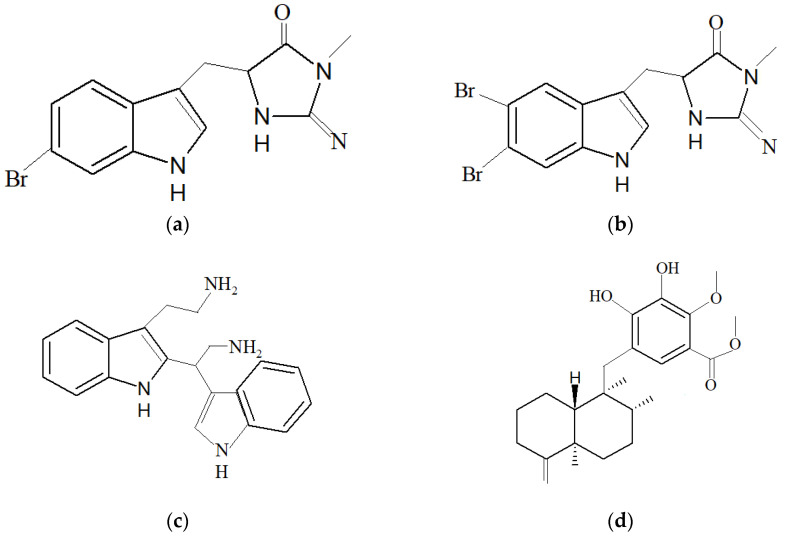
Chemical structures of (**a**) 6-bromo-8,1′-dihydro-isoplycin A; (**b**) 5,6-dibromo-8,1 -dihydro-isoplycin A; (**c**) 6,6′-bis-(debromo)-gelliusine F; and (**d**) 19-methoxy-dictyoceratin A.

*F. reticulata* from Passe Bateau, Mayotte, exhibited antibacterial activity against *Vibrio* sp., which are *Vibrio natrigens* and *Vibrio carchariae* [184]. These bacteria are known for the ability to form biofilm and *V. carchariae* is also a known pathogen that can cause high mortality among fishes. The compounds that exhibit antibacterial activity against *Vibrio* sp. are 6-bromo-8,1′-dihydro-isoplycin A (Figure 6a) and 5,6-dibromo-8,1′-dihydro-isoplycin A (Figure 6b), newly identified compounds. The chemical structures of compounds that were isolated from *F. reticulata* are 6,6′-bis-(debromo)-gelliusine F (Figure 6c), 6-bromo-8,1′-dihydro-isoplycin A and 5,6-dibromo-8,1′-dihydro-isoplycin A.

A new compound, 19-methoxy-dictyoceratin-A (Figure 6d), was identified from the marine sponge *D. elegans* and exhibited anti-cancer properties [182]. The compound was shown to exhibit anti-cancer activity against human cancer cell lines DU145 (prostate cancer cell line), SW1990 (pancreatic cancer cell line), Huh7 (liver cancer cell) and PANC-1 (pancreas cancer cell line).

Apart from the marine sponges, some compounds have been identified from the symbionts that co-exist with marine sponges. One such example would be the symbiont *B. subtilis* NMK17, which is associated with the marine sponge *Clathria frondifera* (Bowerbank, 1875) from the Gulf of Mannar, India. In one study, it was found that the partially purified crude extract from *B. subtilis* exhibited anti-cancer activity against breast cancer cell lines (MCF7, MDA-MB-231 and T47D) in a dose-dependent manner [196]. All three cell lines exhibited a reduction in viability of the cell at a concentration as low as 10 µg/mL. Moreover, the extract also exhibited antibacterial activity against both Gram-positive (*Enterococcus faecalis* ATCC 29212 and *S. aureus* ATCC 25923) and Gram-negative (*P. aeruginosa* ATCC 15,442 and *K. pneumoniae* ATCC 13883) bacteria. These bioactivities of *B. subtilis* are attributed to the presence of secondary metabolites such as fatty acids, lipopeptides, macrolactones, polypeptides, polyketides and isocoumarins [196].

**Figure 7:**
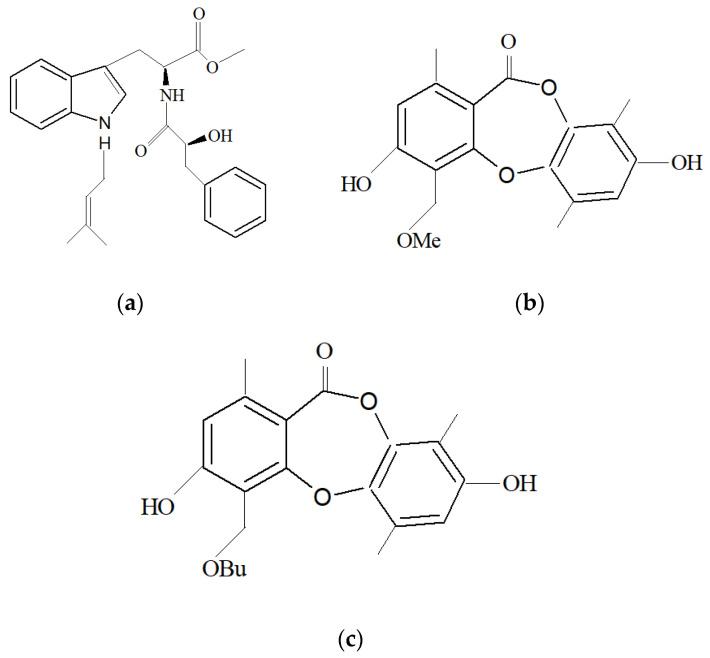
Chemical structures of (**a**) Misszrtine A; (**b**) Botryorhodines I; and (**c**) Botryorhodines J.

Fungi also co-exist with marine sponges as a symbiont. In a study conducted in Xuwen County, China. *Aspergillus* sp. SCSIO XWS03F03, which co-exists as a symbiont with a marine sponge, exhibited anti-cancer activity against HL-60 (promyelocytic leukemia) and LNCap (lymph node from the prostate) cancer cell lines [179]. The compound responsible for the anti-cancer activity was identified to be Misszrtine A (Figure 7a).

Two new compounds belonging to depsidone class were discovered from the fungus *Setosphaeria* sp. SCSIO41009, which co-exists as a symbiont with *Callyspongia* sp., from Xuwen County, China [189]. The compounds Botryorhodines I (Figure 7b) and Botryorhodines J (Figure 7c) were shown to exhibit antifungal activities against phytopathogenic fungi *C. asianum* and *C. acutatum*.

Another example of compounds from marine sponge symbionts that have showcased bioactivity is *Cyanobacteria*, which coexists with *Petrosia ficiformis* (Poiret, 1789) from the Mediterranean region. There were eight strains of *Cyanobacteria* isolated from *P. ficiformis*: *Cyanobium* sp., *Synechococcus* sp., *Pseudabaena* sp., *Leptolyngbya ectocarpi*, *Halomicronema* cf. *metazoicum*, and *Halomicronema metazoicum* [197]. However, only *Pseudabaena* sp. aqueous cell supernatant exhibited apoptosis and inhibitory effects on mitosis on the rat-neuron-like B104 cells, human-neuron-like SH-SY5Y cells and human-epithelial-like (cervical adenocarcinoma) (HeLa) cells.

## 8. Mode of Action

**Figure 8:**
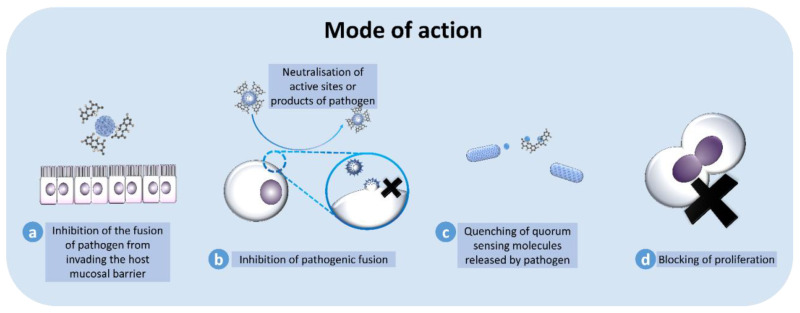
Mode of action of compounds isolated from the marine sponge on various microorganisms: (**a**) inhibition of pathogen from the invading mucosal barrier of the host; (**b**) inhibition of the fusion of pathogenic cells through neutralization of active sites of the pathogen; (**c**) quenching of quorum sensing molecules released by the pathogens; and (**d**) blocking the proliferation of malignant cells.

As mentioned earlier, antimicrobial properties derived from these marine sponges can be utilized in the pharmaceutical industry, which related not only to human diseases, but also applied in many other veterinary areas. Despite various compounds being constantly extracted and identified from the sponges, the understanding of the mode of action of these compounds against the targeted microorganisms is still at the infancy stage. Figure 8 illustrates the possible modes of action of these bioactive compounds against various microorganisms.

Quorum sensing is a way bacterial cells communicate with each other by producing and secreting small signaling molecules known as autoinducers. These molecules accumulate in the environment, which increases the concentration of bacterial cell density [198]. Quorum sensing plays a vital role in biofilm formation and virulence factors by producing phospholipase, hemolysin and protease [199].

In contrast, quorum quenching is a process that interferes with the process of quorum sensing. It could be a technique that can be applied as a novel therapeutic method to control the infection of microorganisms. Quorum quenching is achieved via a few strategies, including (a) amide bond hydrolysis, (b) lactone hydrolysis, and (c) modification of the acyl chain [198].

Amide bond hydrolysis involves enzyme AHL acylase during the degradation of N-acyl-homoserine lactone (AHL). The degradation is an irreversible process. AHL will undergo hydrolysis, releasing the compound homoserine lactone and fatty acid. This enzymatic activity has been identified in the acyl chain completed by the enzymes AHL oxidase and reductase [200]. These are prokaryotic and eukaryotic cells, whereby the AHL-acylase are known to be able to hydrolyze long-chain AHLs [200,201]. Lactone hydrolysis is an enzymatic reaction whereby the enzyme AHL lactonase will induce the hydrolysis of the homoserine lactone ring found in the AHL, to generate acyl homoserine [200]. This reaction is similar to pH-mediated lactonolysis; hence, the reaction can be reversed through acidification of the medium. Lactonase activity has been identified in *Bacillus* sp., which co-exists as a symbiont with the marine sponge. Another method that is applied to inhibit quorum sensing is through the modification of a process found only in bacteria. For example, P450 monooxygenase, an AHLase obtained from *B. megaterium,* is known to oxidize fatty acids and *N*-fatty acyl amino acids.

Marine sponge extracts have also been shown to possess the ability to neutralize the active sites of the pathogen, thus inhibiting the fusion of the pathogenic cells to the host cells. For example, an extract obtained from the marine sponge *Siliquariaspongia mirabilis,* containing compounds Mirabamide A, C and D and papuamide A, has been shown to inhibit the fusion of HIV-1 virus through a neutralization process [202]. These compounds interact with the glycoproteins found on the envelope of the virus, thus inhibiting the fusion of the virus to the host cells.

The extracts of marine sponges have also been identified to possess anti-cancer and anti-tumor activities. As seen in Figure 8, the extracts are able to prevent the growth of cancerous cells by preventing the proliferation of these malignant cells through apoptosis [203,204]. Apoptosis is a physiological mechanism that induces the death of the cells to maintain cellular homeostasis. Some marine sponge extracts may induce apoptosis through an intrinsic pathway. The intrinsic pathway is generally triggered when there is enormous intracellular damage, such as DNA damage, oxidative stress and cytokine withdrawal [205]. Marine sponges such as *Ircinia ramose*, *Monanchora* sp. and *Xestospongia* sp. can induce apoptosis of the cancerous cells by chromatin condensation or DNA fragmentation [205]. Another mechanism to induce apoptosis is based on p53 dependent apoptosis [204]. The genes p21, NOXA, PUMA and Bax are increasingly expressed, and cleaved Caspace-9 and cleaved Caspase-3 also increases, thus resulting in the apoptosis of cancerous cells.

## 9. Conclusions

With the advancement of technology, many sponge species from the poles to the tropics have been tested for their bioactivities. These compounds and extracts have showcased a wide range of potency, ranging from strong to moderate antimicrobial activity towards various pathogens, including antibiotic-resistant bacteria strains, and viruses such as HIV as well as anti-fouling activities. Despite numerous tests being conducted on human-based pathogens, it is also necessary to focus on fish pathogens, such as WSSV and tilapia lake virus, and fungal strains such as *Icthyophonus*, which also causes trouble in the aquaculture industry. However, before these compounds can be commercialized for human or animal applications, in-depth investigations need to be conducted on the toxicity of these extracts and compounds on animal cell lines as well as on the mechanism of these compounds that acts on living cells. Most importantly, the metabolites isolated from marine sponges are only available in a very small volume from the sponge extracts. To overcome this issue, it is necessary to identify methods to synthesize these beneficial compounds on large scale for commercial purposes. Furthermore, the compounds obtained from marine sponges need to be further studied to understand their mode of action against various pathogens, thus elucidating the effect of these compounds at the gene and protein level and their efficacy on pathogenic microorganisms. Only through a series of stringent investigations will new and effective drug agents be developed to mitigate current issues and provide favorable outcomes to stakeholders.

## Figures and Tables

**Table 1:** Compounds isolated from marine sponges and their bioactivity.

Marine Sponge	Compound	Bioactivity	References
***Agelas oroides*** (**Schmidt, 1864**) **associated with *Streptomyces* sp. SBT345**	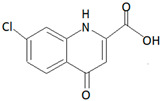 Compound quinolone ageloline A	Inhibitory activity towards *Chlamydia trachomatis* and cytotoxic activity against leukemia cells HL-60	[16,17]
***Dysidea avara*** (**Schmidt, 1862**)	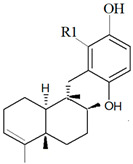 6′-Hydroxy avarol (R1: OH) 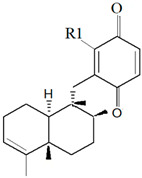 3′-hydroxy avarone (R1: OH)	Antiviral activity against Human Immunodeficiency virus (HIV)	[18,19]
***Hamigera tarangaensis*** (**Bergquist and Fromont, 1988**)	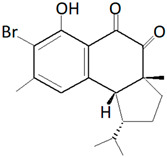 Compound Hamigeran B	Antiviral activity against herpes and polio virus	[20,21]
***Leuconia nivea*** (**Grant, 1826**) **Synthesized by the symbiont bacteria** ***Microbulbifer* sp.**	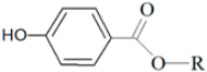 Natural paraben (R:-(CH_2_)_9_ –(CH_3_)	Antibacterial activity against *Staphylococcus aureus*	[17,22]

**Table 2:** Compounds extracted from marine sponges and the chemical structure of the compounds.

Marine Sponge	Classification	Compound	Chemical Structure	References
***Agelas oroides*** (**Schmidt, 1864**)	Bromopyrrole alkaloid	Oroidin	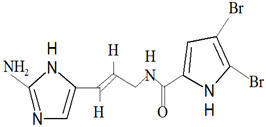	[3,45]
***Discodermia kiiensis*** (**Hoshino, 1977**)	Peptide	Discodermin A	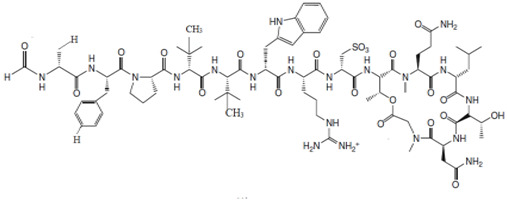	[46]
***Jaspis stellifera*** (**Carter, 1879**) ***Stelleta tenuis*** (**Lindgren, 1897**)	Triterpene	Stelletin A	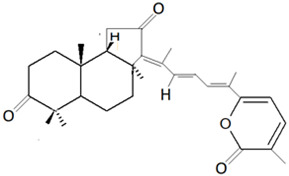	[47]
***Haliclona* sp.**	Polycyclic, β-carboline-derived alkaloid	Manzamine A	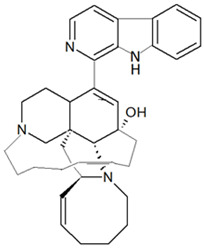	[48,49]
***Luffariella variabilis*** (**Polejaeff, 1884**)	Sesterterpene	Manoalide	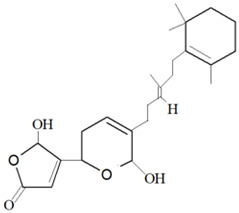	[50]

**Table 3:** Marine sponges and extracted compounds having antimicrobial activity.

Bioactivity	Marine Sponge	Compound	Properties	References
**Antibacterial**	*Axinella donnani* (Bowerbank, 1873)	Lectin	High potency in anti-biofilm activity against biofilm-producing *S. aureus* and able to inhibit the bacterial cells of *S. aureus*.	[95]
	*Clathria compressa* (Schmidt, 1862)	Clathric acid	Able to inhibit growth of methicillin-resistant *S. aureus* ATCC 33,591 (MRSA), *S. aureus* ATCC 6538P and vancomycin-resistant *S. aureus* (VRSA).	[96]
	*Siliquariaspongia* sp. (Hoshino, 1981)	Motualevic acid A Motualevic acid B Motualevic acid E Motualevic acid F	Compounds motualevic acid A and B exhibited potent activity against *S. aureus* at a concentration of 10 µg. Motualevic acid A exhibited antibacterial activity against MRSA at 10 µg. Motualevic acid E exhibited weak antibacterial activity against *S. aureus* at 50 µg. Motualevic acid F exhibited potent antibacterial activity against both *S. aureus* and MRSA at concentrations of 2 µg and 5 µg, respectively.	[97]
**Antiviral**	*Aaptos aaptos* (Schmidt, 1864)	2-isopropyl-10-methoxyimidazo [4,5,1-*ij*]pyrido[2,3,4-*de*]quinolone. 2-(phenethyamino)demethyl(oxy)aaptamine. 3-(isopentylamino)demthyl(oxy)aaptamine.	The compounds 2-isopropyl-10-methoxyimidazo[4,5,1-*ij*]pyrido[2,3,4-*de*]quinolone, 2-(phenethyamino)demethyl(oxy)aaptamine and 3-(isopentylamino)demthyl(oxy)aaptamine inhibited HIV-1 virus replication by 27.7%, 88% and 72.3%, respectively.	[98]
	*Dactylospongia metachromia* (de Laubenfels, 1954)	Metachromin A	Inhibit the Hepatitis B viral production significantly at an EC50 of 0.8 µM.	[99]
	*Stachybotri* sp. HH1 ZSDS1F1-2 fungal strain from unknown marine sponge	Stachybogrisephenone B Grisephenone A 3,6,8-trihydroxy-1-methylxanthone	Antiviral activity against the intestinal virus Enterovirus 71 (EV71).	[100]
**Antifungal**	*A. aaptos*	Methylethylketal derivative of dimethyl(oxy)aaptamine 2-(phenethyamino)demethyl(oxy)aaptamine. 3-(isopentylamino)demthyl(oxy)aaptamine	The compound methylethylketal derivative of dimethyl(oxy)aaptamine exhibited antifungal activity against *Candida parapsilosis*. Compound 2-(phenethyamino)demethyl(oxy)aaptamine exhibited antifungal activity against *C. albicans, C. parapsilosis, Cryptococcus neoformans, Trichophyton rubrum* and *Microsporum gypeseum* at 32, 64, 32, 4, and 16 µg/mL, respectively. 3-(isopentylamino)demthyl(oxy)aaptamine exhibited antifungal activity against *C. neoformans, T. rubrum*, and *M. gypeseum* with MIC of 64, 8, and 32 µg/mL, respectively.	[98]
	*Hippospongia* sp. (Schulze, 1879)	*epi*-ilimaquinone	The compound exhibited potent antifungal activity against amphotericin-resistant *C. albicans* with a MIC value of 125 µg/mL.	[101]
	*Xestospongia exigua* (Kirkpatrick, 1900)	Xestodecalactone B	The extract was able to inhibit the growth of *C. albicans*.	[102]
**Anti-parasite**	New Caledonian deep sea sponge *Verongula rigida* (Esper, 1794)	Meroterpenes Alisiaquinone A Alisiaquinone B Alisiaquinone C Alisiaquinol 11-hydroxyaerothionin Purealidin B Aeroplysinin-A	Exhibited anti-malarial activity against chloroquinone-resistant *Plasmodium falciparum* strain. 11-hydroxyaerothionin exhibited leishmanicidal activity against *Leishmania*. Compound purealidin B showcased anti-malarial activity against *P. falciparum* strain. Compound aeroplysinin-A inhibit the growth of the parasite *Trypanosoma cruzi*. All the compounds with anti-parasitic activity were found to be bromotyrosines.	[103,104]

**Table 4:** Marine sponge species and their application in the aquaculture industry.

Bioactivity	Marine Sponge	Compound	Application	Reference
Antibacterial	*Acanthella kletra* (Pulitzer-Finali, 1982)	Axisonitrile-3	Antibacterial activity against *Vibrio harveyi* at concentration 250 µg/mL.	[142]
	*Callyspongia diffusa* (Ridley, 1884)	9,12-Octadecadienoic acid	Moderate antibacterial activity against *Vibrio* *fluvialis, V. harveyi* and *Vibrio vulnificus*. Potent activity against *Vibrio anguillarum*.	[143]
	*Hexadella* sp. (Topsent, 1896)	11-*N*-methyl-moloka’iamine 11-*N*-cyano-11-*N*-methylmoloka’iamine Kuchinoenamine	Moderate activity against *Aeromonas hydrophila* at concentration 100 µg/diameter 6.5 mm disk.	[144]
	*Streptomyces tirandamycinicus* from unknown sponge, coast of Wenchang City, Hainan Province of China	Tirandamycin A and B	Both tirandamycin A and B exhibit potent antibacterial activity against *Streptococcus agalactiae* HNe0 at MIC values of 2.52 and 2.55 µg/mL, respectively.	[145]
Antifungal	*Negombota magnifica* (Keller, 1889)	Latrunculin B	Potent activity against fish fungal pathogens *Exophiala salmonis, Branchiomyces demigrans* and *Saprolegnia* sp.	[146]
Antiviral	*Callyspongia* sp.	Polyhydroxy isocopalane	Strong antiviral activity against white spot syndrome virus (WSSV) at concentration 60 mg/L.	[147]
Antifouling	*Acanthella cavernosa* (Dendy, 1922)	Kalihones A, E, O-T 10-*epi*-kalihinol X,I 10β-formamidokalihinool-A 10β-formamido-5β-isothiocyanatokalihinool-A	Inhibited the larvae settlement of *Balanus amphitrite*.	[148]
	*Agelas* sp. (Duchassaing and Michelotti, 1864)	Agelasine D	Prevented the settlement of larvae *Balanus improvises*. The inhibition was dose-dependent manner, ranging from concentration 0.024 µM to 24 µM.	[149]
	*Cymbastela hooperi* van Soest (Desqueyroux-Faùndez, Wright and König, 1996)	Diterpene isonitrile	Inhibit the settlement of the diatom *Nitzschia closterium* at concentration 10 µg/mL.	[142]
	*Haliclona* sp.	Poly 3-alkylpyridinium salt Saraine-1 Haminol-2 Haminol-4	Poly-3 alkylpyridinium salt was potent to inhibit the settlement of the *Amphibalanus* (*Balanus*) *amphitrite* at EC_50_ 0.19 µg/mL. Saraine-1 and haminol-2 equally potent in inhibiting the settlement of the larvae of *A. amphitrite* with an EC_50_ of 0.53 and 0.28 µg/mL, respectively. Haminol-2 and haminol-4 completely inhibited the settlement at the concentration of 10 µg/mL.	[150]
	*Ircinia oros* (Schmidt, 1864)	Mixture of Ircinin I Ircinin II	Anti-feedant activity against predator fish *Thalassoma pavo*. 0% pellets with the mixture consumed by the fishes. Showcased potent inhibition towards the macroalgae *Enteromorpha intestinalisi, Ulva lactuca* and *Sargassum muticum*.	[151]
	*Ircinia spinosula* (Schmidt, 1862)	Hydroquinone-A Hydroquinone-B	Hydroquinone-A exhibited moderate inhibition on *E. intestinalis* macroalgae and strong inhibition on *U. lactuca* and *S. muticum* macroalgae. Hydroquinone-B showcased poor anti-settlement activity on all three macroalgae.	[151]
	*Ircinia variabilis* (Schmidt, 1862)	Variabilin	Anti-feedant activity against predator fish *T. pavo*. 5% pellets with the mixture consumed by the fishes.	[151]
	*Xestospongia testudinaria* (Lamarck, 1815)	Aragusterol B 21-*O*-octadecanoyl-xestokerol A	The compounds exhibited potent anti-adhesion on biofilm against *Pseudoalteromonas* sp. D41 and TC8 and *Polaribacter* sp. TC5. Aragusterol-B exhibited EC_50_ values of 23, 20 and 60 µM on *Pseudoalteromonas* sp. D41, TC8 and *Polaribacter* sp. TC5, respectively. 21-*O*-octadecanoyl-xestokerol A had EC_50_ values of 25, 10 and 36 µM on *Pseudoalteromonas* sp. D41, TC8 and *Polaribacter* sp. TC5, respectively	[152]

**Table 5:** Summary of marine sponge extracts and compounds with bioactivity for veterinary commodities.

Marine Sponge	Compound	Applications	References
***Aplysina thiona*** (**Laubenfels, 1930**)	Fistularin 3 11-ketofistularin 3	Inhibition of feline leukemia virus with an ED_50_ 22 µM for Fistularin 3 and 11-ketofistularin 3 at 42 µM.	[165]
***Axinella* sp.** (**Schmidt, 1862**)	Halichondrin B	Displayed antimitotic activity as it is capable of binding to bovine tubulin and inhibited microtubule assembly in vitro.	[166]
***Cinachyrella* sp.** (**Wilson, 1925**)**, *Haliclona* sp., and *Petromica citrine*** (**Muricy, Hajdu, Minervino, Madeira and Peixinho, 2001**)	Aqueous extract	Showcased antibacterial activity against mastitis-causing Coagulase-negative *Staphylococci.*	[167]
***Monanchora* sp.** (**Carter, 1883**)	NOR-Batzelladine L	Antiviral activity against avian metapneumovirus due to the presence of compound NOR-Batzelladine L.	[168,169]
***Phoriospongia* sp.** (**Marshall, 1880**)	Phorioadenine A	Antiparasitic effect against *Haemonchus contortus* larval development, an important nematode parasite of the ruminant industry.	[170]
***Plakortis halichondrioides*** (**Wilson, 1902**)	Plakortides	Positive action in activating Ca^2+^ pumping activity of canine-derived cardiac sarcoplasmic reticulum, leading to cardiac relaxation.	[171]

**Table 6:** List of compounds with positive bioactivity discovered from marine sponges and their associates from 2018 to 2021.

Marine Sponge and Associates	Location	Compound	Classification	Bioactivity	Reference
***Acanthostrongylophora ingens*** (**Thiele, 1899**)	South Sulawesi, Indonesia	*epi-*tetradehydrohalicyclamine B and tetradehydrohalicyclamine B, acanthocycloamine A, halicyclamine B chloromethylhalicyclamine B and diketopiperazines.	Alkaloids	Antibacterial activity, reduce the production of amyloid β-42, and antikinase activity.	[178]
***Aspergillus* sp., a marine fungus associated with marine sponge** (**not specified**)	Xuwen County, China	Misszrtine A	Alkaloids	Anti-cancer activity against HL-60 and LNCap cells.	[179]
***Cacospongia mycofijiensis*** (**Kakou, Crews and Bakus, 1987**)	‘Eua, Kingdom of Tonga	Zampanolides B, C and D	Macrolide	Anti-proliferative and anti-mitotoic activities with microtubule stabilizing activity.	[180]
***Dactylspongia elegans* T3** (**Thiele, 1899**	North Sulawesi, Indonesia	Nakijiquinone V, illimaquinone, smenospongine and dyctioceratine C.	Sesquiterpene aminoquinone, sesquiterpene quinones and sesquiterpene hydroquinone.	Antibacterial activity.	[181]
***Dactylspongia elegans* T3** (**Thiele, 1899**	Yongxing Island in the South China Sea	19-methoxy-dictyoceratin-A	Sesquiterpene quinones	Anti-cancer activity against the human cancer cell lines DU145, SW1990, Huh7, and PANC-1	[182]
***Haliclona gracilis*** (**Miklucho-Maclay, 1870**)	Shikotan Island	Gracilosulfates A, B, C, D, E, F and G	Steroid	Anti-tumor activity against human prostate cancer.	[183]
***Fascaplysinopsis reticulata*** (**Hentschel, 1912**)	Passe Bateau, Mayotte	6-bromo-8,1′ -dihydro-isoplysin A and 5,6-dibromo-8,1′ -dihydro-isoplysin A	Tryptophan derived alkaloids	Antibacterial activity against *Vibrio* sp.	[184]
***Haliclona* sp.** (**Grant, 1836**)	Mayotte	Osirisynes G, H and I	Long-chain highly oxygenated polyacetylenes	Enzyme-inhibitory activity against proteasome kinase.	[185]
***Monascus* sp.** (**Tiegh, 1884**)**, a marine fungus associated with the marine sponge *Clathria frondifera*** (**Bowerbank, 1875**)	Gulf of Mannar	Monacolin X	Polyketide	Anti-proliferative and anti-migratory activities against human breast cancer cell lines.	[186]
***Mycale aff. nularosette*** (**Hoshino, 1981**)	Miyagi, Japan	Mycalolide A, mycalolide B and 38-hydroxymycalolide B	Macrolide	Actin depolymerization resulting in incomplete cytokinesis.	[187,188]
***Setosphaeria* sp.** (**Leonard and Suggs, 1974**)**, marine fungus associated with the marine sponge *Callyspongia* sp.** (**Duchassaing and Michelotti, 1864**)	Xuwen County, China	Botryorhodines I and J	Depsidones	Moderate antifungal activities against the phytopathogenic fungi *Colletotrichum asianum* and *Colletotrichum acutatum*.	[189]
***Stylissa carteri*** (**Dendy, 1889**)	Indonesia		Flavonoid, triterpenoid and steroid	Anti-cancer activity against breast cancer MDA MB 231 cell line.	[190]
